# Essential oils sensory quality and their bioactivity against the mosquito *Aedes albopictus*

**DOI:** 10.1038/s41598-018-36158-w

**Published:** 2018-12-14

**Authors:** S. Bedini, G. Flamini, R. Ascrizzi, F. Venturi, G. Ferroni, A. Bader, J. Girardi, B. Conti

**Affiliations:** 1 Department of Agriculture, Food and Environment – University of Pisa, Pisa, Italy; 2 Department of Pharmacy – University of Pisa, Pisa, Italy; 3 Faculty of Pharmacy, Umm Al-Qura University, Makkah, Saudi Arabia

## Abstract

Repellents are a main tool to prevent the outbreak of mosquito-borne diseases that represents a threat for millions of people worldwide. Plant-based products are very promising, low-toxic and eco-friendly alternative to synthetic repellents. Here, we performed an olfactory screening of the essential oils (EOs) of *Artemisia verlotiorum* Lamotte (Asteraceae), *Lavandula dentata* L. (Lamiaceae), and *Ruta chalepensis* L. (Rutaceae) for their possible use as ingredients in topical repellents. The EOs smell profiles were then matched with their repellence against the mosquito *Aedes albopictus* (Skuse) (Diptera Culicidae). To obtain a more complete bioactivity description, we also tested the EOs oviposition deterrence and the larvicidal activity. The best smell profile was associated with *A*. *verlotiorum* EO, while *R*. *chalepensis* EO showed the lowest overall pleasantness. All the EOs had a significant activity as skin repellent against *Ae*. *albopictus*, deterred the oviposition in the field, and exerted a clear larvicidal activity. Beside the best smell profile, *A*. *verlotiorum* EO showed also the longest lasting repellent effect, assuring the complete protection of the treated skin against *Ae*. *albopictus* for a time 60% longer than the synthetic repellent DEET.

## Introduction

The Asian tiger mosquito, *Aedes albopictus* (Skuse) (Diptera Culicidae) is ranked among the most invasive mosquito species in the world^1^. Native to the tropical and subtropical areas of Southeast Asia, in recent time, *Ae*. *albopictus* has spread across many countries including Europe, North and South America, and Africa^2^. Besides its aggressive daytime biting behaviour, the medical importance of *Ae*. *albopictus* is due to its ability to transmit many human pathogens and parasites (e.g. yellow fever, dengue fever, West Nile, Japanese encephalitis, St. Louis encephalitis, chikungunya viruses, filarial nematodes)^3^.

Since no vaccines or drugs are available for most of the pathogens carried by *Ae*. *albopictus*, the control of mosquitoes remains the essential tool for the prevention of the transmission of many of them^4^. To date, the control of adult mosquitoes commonly relies on the use of synthetic insecticides and repellents, mainly belonging to organophosphates and pyrethroids^5^, but treatments with such chemicals are expensive, show scarce efficacy and have a strong environmental impact^6^. Also *N*,*N*-Diethyl-*meta*-toluamide (DEET), a synthetic compounds that is the most common active ingredient in insect repellents, has been associated to relevant human health risks^7^. For these reasons, alternative natural insecticides and repellents are now very appreciated by consumers. Essential oils (EOs) of aromatic plants are considered among the most promising alternative to synthetic chemicals^8,9^. EOs are generally recognized as environmental friendly, easily biodegradable, minimally toxic to mammals and have shown repellent^10,11^, toxicant^12–14^, oviposition deterrent, growth and/or reproduction inhibition^15,16^ activities against different mosquito species. In some cases, the activity of these compounds is higher, or it has longer duration than synthetic chemicals^17,18^.

Because of their properties, EOs have been proposed as active ingredients in mosquito-repellent topical products^19^ as well as in repellent and insecticidal mists sprayed by automatic outdoor systems^20^. However, despite their efficacy, low cost and low toxicity, EOs still do not have a widespread application due to some limitations such as their high volatility, composition variability and strong smell. In fact, the sensory impact of EOs has been reported as one of the most negative aspects of their use^21^. To that regard, to ensure market success as ingredient in EOs-based environmental and topical repellents, EOs should have a high efficacy against the target insect coupled with a pleasant smell for the consumers. To date, however, no specific investigations have been performed to evaluate their bioactivity against the target insects and their impact on human sensorial system.

Lamiaceae, Asteraceae, and Rutaceae are among the main families investigated for their anti-mosquito properties. Given this, in the present research, the EOs, extracted from *Artemisia verlotiorum* Lamotte (Asteraceae), *Lavandula dentata* L. (Lamiaceae), and *Ruta chalepensis* L. (Rutaceae), were evaluated for their efficacy as repellents, larvicides, and oviposition deterrents against *Ae*. *albopictus* together with their impact on human sensorial system.

## Results

### Essential oils smell characterization

**Figure 1:**
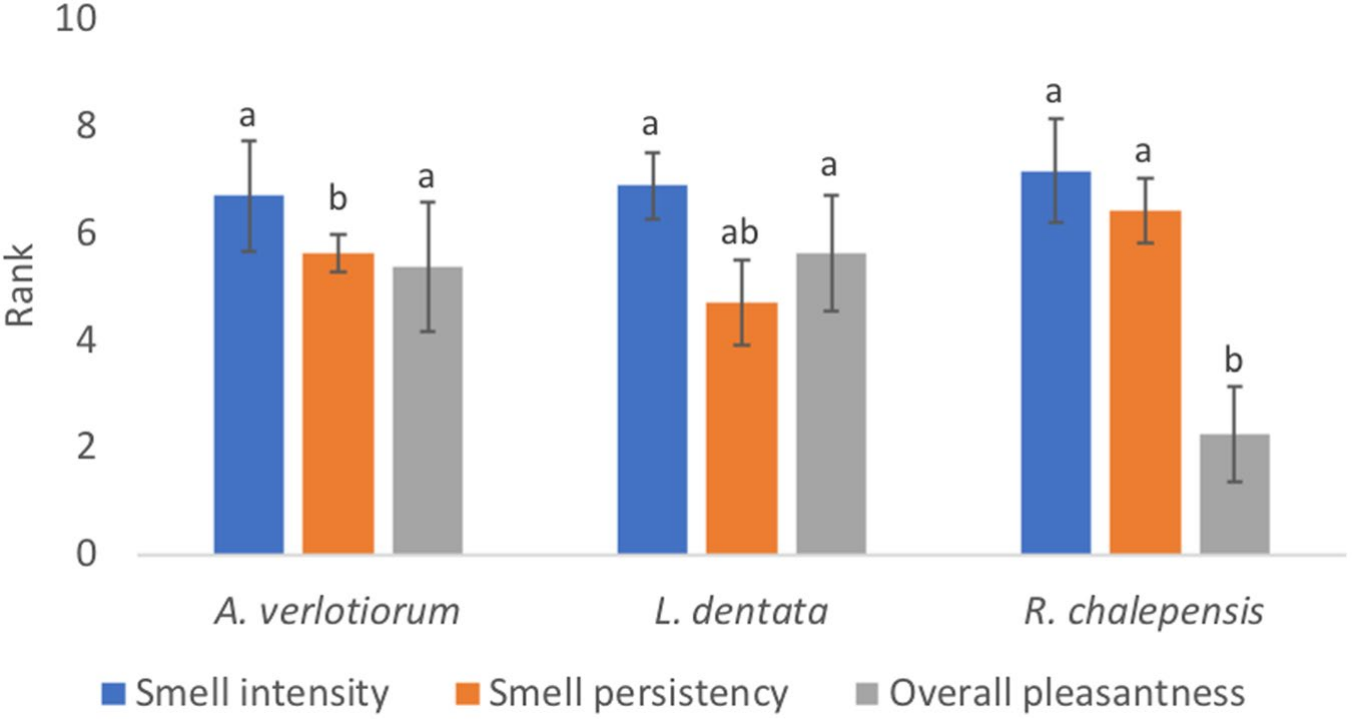
Essential oils sensorial description. *Artemisia verlotiorum*, *Lavandula dentata*, and *Ruta chalepensis* main essential oils (EOs) sensorial descriptors ranked by panel components on a 0-to-10 scale. Histograms represent the mean of the ranks; different letters indicate significant differences (Tukey’s b, *P* ≤ 0.05) for each descriptor among EOs; bars represent the confidence interval.

All the tested EOs were characterised by the same “Smell intensity” (*F*_(2,36)_ = 0.723, *p* = 0.764), while the “Smell persistency” as well as the “Overall pleasantness” resulted significantly different (*F*_(2,36)_ = 4.069, *p* = 0.026; *F*_(2,36)_ = 11.941, *p* < 0.001, respectively). In comparison with the *R*. *chalepensis* EO, *L*. *dentata* EOs showed a lower “smell persistency” associated with a higher “Overall pleasantness” (Fig. 1).

**Figure 2:**
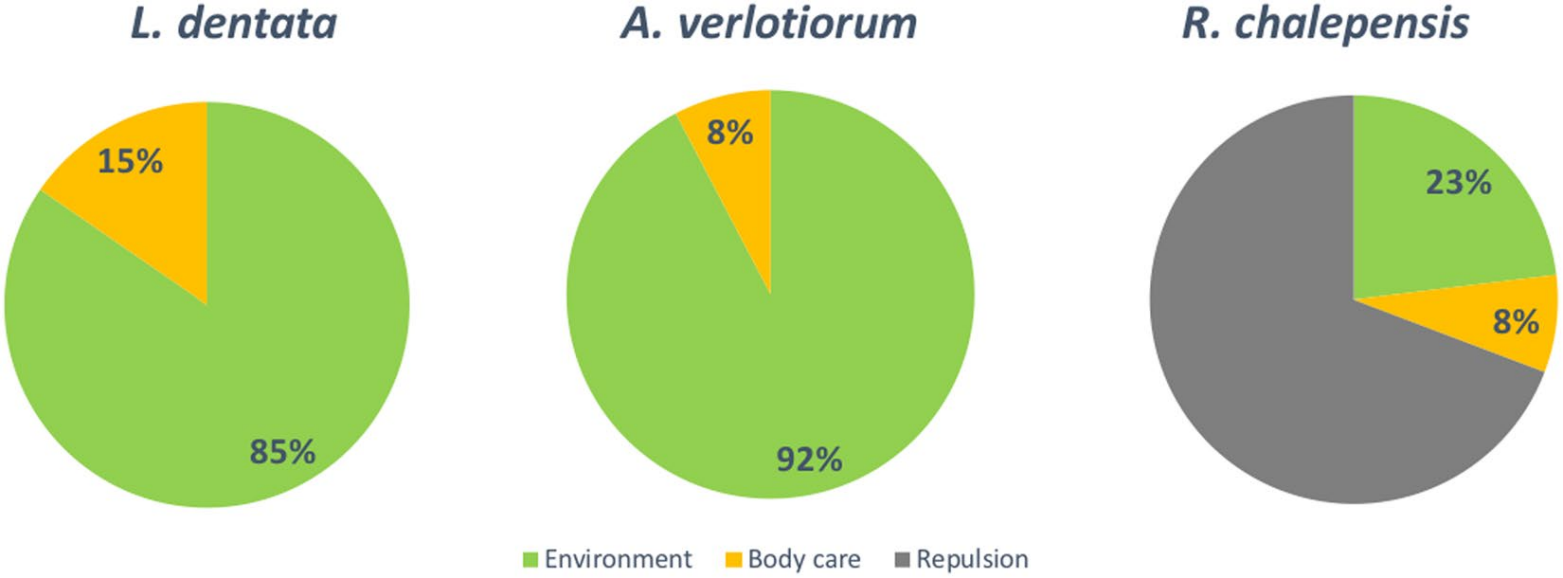
Suggested use of essential oils. Suggested use (Environmental or Body care) of the *Artemisia verlotiorum*, *Lavandula dentata*, and *Ruta chalepensis* essential oils as assessed by panel test. Values represent the percentage of the suggested use.

In this context, the best smell profile was associated with *A*. *verlotiorum* EO, closely followed by the *L*. *dentata* EO. *R*. *chalepensis* EO was characterized by the worst smell profile with the highest number of off-flavours indicated by panellists (Table 1). Most part of the judges (69%) associated the *R*. *chalepensis* EO to bad emotions represented by “Repulsion”, while *A*. *verlotiorum* and *L*. *dentata* EOs were associated with the main possible use in “Environmental protection” followed by “Body care” (Fig. 2).

**Table 1:** Main odours that characterized the smell of *Artemisia verlotiorum*, *Lavandula dentata*, and *Ruta chalepensis* essential oils (EOs).

Odour class/EO	*A*. *verlotiorum*	*L*. *dentata*	*R*. *chalepensis*
**Vegetative odours**	Mint	Mint	
	Sage	Rosemary	
	Chamomile	Herbaceous	
	Citronella		
**Spicy**			Balsamic
			Cocoa
			Sandalwood
**Other**	Honey		
**Off-flavors**		Medicinal	Smoky
		Fresh paint	Resin
			Wet leather
			Animal

### Essential oils chemical analyses

The compositions (compounds with relative abundances >1%) of the essential oils (EOs) are reported in Table 2. The complete composition of the EOs can be found as Supplementary Table S1.

**Table 2:** Chemical composition (%) of the *Artemisia verlotiorum* e *Lavandula dentata* essential oils used in the assays.

Constituents^a^	*LRI*	*L*. *dentata*	*A*. *verlotiorum*	*R*. *chalepensis*
α-pinene	941	1.2	1.2	
camphene	955	1.3		
β-pinene	981	2.7		
myrcene	993		1.9	
limonene	1032	1.9		2.2
1,8-cineole	1035		7.1	
*cis*-sabinene hydrate	1070		1,0	
*cis*-linalool oxide (furanoid)	1076	1.8		
fenchone	1089	26.2		
2-nonanone	1093			56.7
linalool	1101	2.2		
2-nonanol	1101			3.5
2,6-dimethyl phenol	1108		5.2	
*exo*-fenchol	1118	4.2		
chrysanthenone	1127		34.3	
geijerene	1143			1.7
camphor	1145	52.1		
β-pinene oxide	1158		2.2	
borneol	1167	1.4		
2-decanone	1193			1.7
2-nonyl acetate	1237			14.6
perilla aldehyde	1273		1,0	
2-undecanone	1294			12.7
2-undecanol	1308			1.3
β-caryophyllene	1420		12.6	
α-humulene	1456		1.3	
2-undecyl acetate	1475			1.4
γ-muurolene	1477		9.9	
bicyclogermacrene	1495		1.2	
caryophyllene oxide	1581		4,0	
selin-11-en-4-α-ol	1653		3,0	

^a^Chemical constituents ≥ 1%; LRI, linear retention index on DB-5 column.

A total of 25 compounds have been identified in the *L*. *dentata* EO, accounting for up to 99.60% of the total composition. Oxygenated monoterpenes dominated its composition, representing over 90% of the total. Of this chemical class of compounds, camphor and fenchone added up to over 75% of the EO, showing relative abundances of 52.05 and 26.19%, respectively. *Exo*-fenchol follows, with a relative concentration of 4.19%. Finally, among monoterpene hydrocarbons (relative abundance of 7.67%) β-pinene (2.69%) and limonene (1.93%) were the most relevant compounds.

The 96.38% of the EO of *A*. *verlotiorum* was composed by 39 compounds, belonging to the monoterpenes, sesquiterpenes, phenylpropanoids and other non-terpene derivatives chemical classes. As in *L*. *dentata* EO, but with an almost halved relative concentration, the most abundant chemical class of *A*. *verlotiorum* EO was represented by monoterpene hydrocarbons. Accounting for up to 34.27%, chrysanthenone was the most represented compound of this class and of the entire EO composition, followed by 1,8-cineole (7.10%). The sesquiterpene hydrocarbons followed: this chemical class was mainly represented by β-caryophyllene (12.62%, the second most abundant compound in the total EO) and γ-muurolene (9.90%). In this EO, monoterpene hydrocarbons showed an almost halved (4.17%) relative abundance compared to *L*. *dentata* EO, with myrcene (1.89%) and α-pinene (1.19%) as the most represented ones.

A completely different chemical composition was found for *R*. *chalepensis* EO: 19 compounds represented 98.84% of the complete composition, of which 93.40% was composed of non-terpene derivatives. 2-nonanone and the closely related ester 2-nonyl acetate were the most abundant compounds in this EO composition, accounting for up to 56.73 and 14.61%, respectively. 2-undecanone followed, with a relative presence of 12.74%. The monoterpene hydrocarbons chemical class accounted for only 2.82%, with limonene as the most represented one (2.23%), closely followed by sesquiterpene hydrocarbons (2.38%).

### Essential oils oviposition deterrence

**Figure 3:**
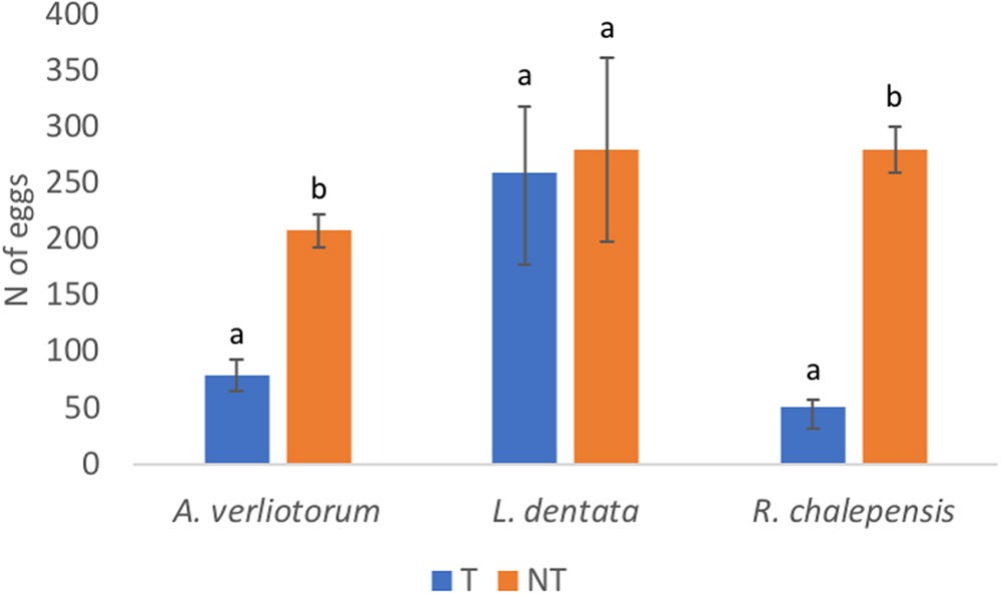
Mosquitoes oviposition deterrence. *Aedes albopictus* oviposition deterrence of *Artemisia verlotiorum*, *Lavandula dentata*, and *Ruta chalepensis* essential oils. Histograms represent the mean total number of eggs laid in 14 days; bars represent standard error; different letters indicate significant differences between treated, T and non-treated, NT ovitraps (Student’s t-test *P* ≤ 0.05).

The tested EOs showed a clear ability to deter *Ae*. *albopictus* oviposition. After 14 days, the mean number of eggs laid on the control Masonite strips were 273.91 ± 21.79 whereas, with the EOs, the number of eggs on the strips ranged from 51.50 ± 5.68 to 259.50 ± 41.01 (Fig. 3). The reduction of oviposition was significant for *A*. *verlotiorum* and *R*. *chalepensis* EOs, (*F*_(1,6)_ = 10.248, *p* = 0.019; *F*_(1,6)_ = 118.418, *p* < 0.001, respectively) but not for *L*. *dentata* EO (*F*_(1,6)_ = 0.162, *p* = 0.701). OAI values were 0.52 ± 0.07, −0.08 ± 0.00, and −0.69 ± 0.02 for *A*. *verlotiorum*, *L*. *dentata* and *R*. *chalepensis* EOs respectively, and confirmed the deterrence of *A*. *verlotiorum* and *R*. *chalepensis* EOs with no significant difference between the two EOs (Kruskal-Wallis pairwise comparison, *p* = 0.506).

### Essential oils larvicidal activity

All the tested EOs showed a clear larvicidal activity against *Ae*. *albopictus*. The median lethal concentration (LC_50_) of EOs, calculated by probit regression showed that *R*. *chalepensis* EO resulted the most toxic one with LC_50_ = 93.60 μL L^−1^ while, *A*. *verlotiorum* and *L*. *dentata* EOs LC_50_ values were 324.00 and 602.80 μL L^−1^, respectively (Table 3). The relative median potency (RMP) analysis of probits showed that *R*. *chalepensis* EO was significantly more effective than *A*. *verlotiorum* and *L*. *dentata* EOs (*R*. *chalepensis vs A*. *verlotiorum*, RMP: 0.232, CI: 0.032–0.597; *R*. *chalepensis vs L*. *dentata*, RMP: 0.194, CI: 0.025–0.497), while the difference between *A*. *verlotiorum* and *L*. *dentata* EOs was not significant (*A*. *verlotiorum vs L*. *dentata*, RMP: 0.839, CI: 0.401–1.613).

**Table 3:** Larvicidal activity of the essential oils of *Artemisia verlotiorum*, *Lavandula dentata*, and *Ruta chalepensis* against *Aedes albopictus*.

	*A*. *verlotiorum*	*L*. *dentata*	*R*. *chalepensis*
LC_50_^a^ (CI)	324.002 (279.721–397.777)	602.800 (442.119–1001.284)	93.601 (82.700–105.238)
LC_95_^b^ (CI)	957.048 (667.151–2006.186)	9509.296 (3873.041–51145.558)	217.993 (174.019–332.812)
Slope ± SE	3.497 ± 0.641	1.373 ± 0.217	4.480 ± 0.751
Intercept ± SE	−8.779 ± 1.559	−3.817 ± 0.522	−8.831 ± 1.493
χ^2^ (df)	0.71 (2)	4.42 (4)	4.69 (3)
*P*	0.700	0.246	0.196

^a^Concentration of the extract that kills 50% of the exposed larvae; ^b^Concentration of the extract that kills 95% of the exposed larvae. Data are expressed as μL L^−1^; CI, Confidence Interval; (df), degrees of freedom; *P*, Pearson Goodness-of-Fit Test.

### Essential oils repellent activity

The arm in cage tests showed that the dose of *R*. *chalepensis* EO, able to repel females of *Ae*. *albopictus*, was lower than the *A*. *verlotiorum* and *L*. *dentata* ones. The median effective dose (ED_50_) of EOs, showed that *A*. *verlotiorum* EO was the least effective of the three EOs, with ED_50_ value of about 0.8 nL cm^−2^ of skin, while ED_50_ of *L*. *dentata* and *R*. *chalepensis* were 0.4 and 0.2 nL cm^−2^ of skin, respectively (Table 4). RMP analyses of probits showed that the differences between *R*. *chalepensis* and *A*. *verlotiorum* (RMP: 0.274, CI: 0.135–0.555), and between *L*. *dentata* and *A*. *verlotiorum* (RMP: 0.526, CI: 0.277–0.955) are significant. No statistically significant differences were observed between *R*. *chalepensis* and *L*. *dentata*: (RMP: 0.520, CI: 0.273–1.038). On the contrary, the duration of the effect was longer for *A*. *verlotiorum* compared to *L*. *dentata* and *R*. *chalepensis* EOs, with a statistically significant difference (Breslow test: *χ*^*2*^ = 5.142, *P* = 0.02; *χ*^*2*^ = 13.588, *P* < 0.001, respectively). Consistently, the duration of the residual protective effect was longer for *A*. *verlotiorum* with respect to *L*. *dentata* and *R*. *chalepensis* EOs with differences, considering all the doses tested, statistically significant between *A*. *verlotiorum* and *R*. *chalepensis* EOs (Breslow test: *χ*^*2*^ = 6.063, *P* = 0.014). At the intermediate dose of 0.04 μL cm^−2^ skin, the median complete protection time (MCPT) of the EOs, ranged from 16 to 10 min, for *A*. *verlotiorum* and *R*. *chalepensis*, respectively, while, the median duration of the complete protective effect of DEET was 10 minutes (Table 5). At the same dose (0.04 μL cm^−2^ skin), the median residual protection time (MRPT) were 51, 36, and 45 min for *A*. *verlotiorum*, *L*. *dentata*, and *R*. *chalepensis*, respectively, while the residual protection of DEET ended after 70 minutes (Table 5). The complete set of repellence data (mean percentage of repellence ± standard deviation) can be found as Supplementary Tables S2, S3 and S4.

**Table 4:** Repellence of the essential oils of *Artemisia verlotiorum*, *Lavandula dentata*, and *Ruta chalepensis* against females of *Aedes albopictus*.

	*A*. *verlotiorum*	*L*. *dentata*	*R*. *chalepensis*
ED_50_^a^ (CI)	0.0008 (0.0005–0.0013)	0.0004 (0.0003–0.0006)	0.0002 (0.0001–0.0003)
ED_95_^b^ (CI)	0.0271 (0.0100–0.1402)	0.0114 (0.0057–0.0310)	0.0574 (0.0265–0.1670)
Slope	1.061 ± 0.066	1.145 ± 0.054	0.679 ± 0.034
Intercept	3.307 ± 0.245	3.872 ± 0.205	2.487 ± 0.111
χ^2^ (df)	8.25 (4)	8.42 (4)	6.88 (4)
*P*	0.083	0.077	0.226

^a^Dose of the extract that repels 50% of the exposed insect; ^b^Dose of the extract that repels 95% of the exposed insect. Data are expressed as µL cm^−2^ of skin; CI, Confidence Interval; (df), degrees of freedom; *P*, Pearson Goodness-of-Fit Test.

**Table 5:** Median complete protection time (MCPT) and Median residual protection time (MRPT) of *Artemisia verlotiorum*, *Lavandula dentata*, and *Ruta chalepensis* essential oils against *Aedes albopictus* females.

EO	Dose^a^	MCPT	CI	MRPT	CI
*A*. *verlotiorum*	0.01	2.33	2.13–2.53	31.00	11.68–50.32
	0.02	11.00	10.46–11.54	43.00	32.27–53.74
	0.04	16.34	13.33–19.35	51.00	42.70–59.30
	0.08	21.03	18.26–23.80	63.00	25.79–100.21
*L*. *dentata*	0.01	1.50	0.19–2.81	22.00	19.85–24.15
	0.02	10.25	4.88–15.62	41.00	38.85–43.15
	0.04	11.33	8.56–14.10	36.00	26.20–45.80
	0.08	11.50	0.00–23.17	75.00	21.32–128.68
*R*. *chalepensis*	0.01	1.25	1.07–1.43	40.00	29.22–50.78
	0.02	2.20	0.70–3.70	49.00	44.71–53.29
	0.04	10.22	0.00–28.75	45.00	0.90–89.10
	0.08	16.65	0.00–46.49	94.00	66.09–121.91
DEET	0.04	10.00	3.99–16.00	70.00	62.46–77.54

^a^μL/cm^2^ skin; CI, confidence interval. Data are expressed as min.

## Discussion

Personal protection by mosquito repellents is a key measure for the control of several life-threatening vector-borne diseases and synthetic compounds (i.e. DEET, DEPA, IR3535, and Picaridin) are commonly used as active ingredient in insect repellent products^22^. However, an increasing number of evidences suggest that such synthetic repellents may trigger undesirable hazardous interactions with biological systems, with a potential to generate harmful effects^7,22,23^. As an alternative, over the last 50 years, many plant species have been screened as potential sources of repellents and insecticides. Even if, for most plant-derived products, the supposed low environmental and mammalian toxicity have not been sufficiently investigated^24,25^, in some cases, such plant-derived repellents are utilized as active ingredient in commercial repellent. This is the case of the *p*-menthane-3,8-diol (PMD), extracted from the lemon eucalyptus, *Corymbia citriodora* Hook (Mirtaceae)^22^. Nonetheless, for their use as active ingredients in topical or environmental spray products, EOs should not only be persistent and effective against mosquitoes but they also should be sensorially well accepted by consumers. To the best of our knowledge, there are no studies coupling the bioactivity of the EOs against insects and their sensory impact on customers. In fact, the sensory impact of EOs has been reported as one of the most negative aspects of their use^21^.

Our results showed that *A*. *verlotiorum*, *L*. *dentata*, and *R*. *chalepensis* EOs are toxic and repellent against *Ae*. *albopictus* but very different for pleasantness and evocative effects to the human olfactory system. The experimental results showed that EOs with different chemical composition have different smell properties that can influence the consumer acceptability.

The panel test identified *A*. *verlotiorum* EO as the most pleasant-smelling one. The significant relative abundance of chrysanthenone in its composition is probably the reason of its panellists’ preference: Lawrence^26^ described it as a slightly oily-floral aroma suitable for aromatic industry. Together with 1,8-cineole and β-caryophyllene, it may be responsible for the vegetal fresh odour described by the panel^27^. Chrysanthenone, indeed, has been reported as a strong but very pleasant smell contributor in the EO of another species of the same genus (*A*. *herba-alba*) by Rekkab *et al*.^28^. This oxygenated monoterpene aroma contribution has also been described as a sweet, floral scent in the EOs extracted from two other species of the Compositae family: *Laggera tomentosa* Sch. Bip. ex Oliv. et Hiern (Asteraceae) and *Otanthus maritimus* (L.) Hoffmanns. & Link (Asteraceae)^29,30^. A relevant contribution to *A*. *verlotiorum* EO aroma is also that of β-caryophyllene, which is commonly referred to as a “green” flavour contribution: its woody-spicy, dry and clove-like aroma is responsible for the aroma of leafy green vegetables^31^. In our tests, the *A*. *verlotiorum* EO pleasantness score was closely followed by *L*. *dentata* EO, characterized by “Medicinal and Fresh paint” off-flavours that could be explained by the presence of camphor and fenchone as main chemical components. These compounds can, indeed, be associated with an intense pharmaceutical and chemical odour. In the *L*. *dentata* EO, the well-known warm, minty and ethereal aroma of camphor is coupled with the fenchone contribution^32^. This oxygenated monoterpene has a powerful balsamic, almost pine-like, and spicy notes^33^. On the contrary, the unpleasantness of *R*. *chalepensis* EO may due to the presence of 2-nonanone, a non-terpene derivative which determines a characteristic rue odour. Its strong fatty smell, with a very low odour threshold, is one of the most important contributors to soft mould-ripened cheeses and Blue cheese aromas^27^. The closely-related ester, 2-nonyl acetate, exhibits a “fruitier” but unpleasant nuance, involved in the smell of ripening banana. It is, therefore, not advisable to use it in topic formulations, as its odour is strong and unpleasant^34^.

The chemical composition of the *R*. *chalepensis* EO, tested in this work, showed some differences respect to the EOs from the same plant species tested in previous works against mosquitoes. *R*. *chalepensis* EO from Turkey, tested against *Aedes aegypti* L. (Diptera Culicidae) for biting deterrence, repellence, and larvicidal activities^35^, showed different percentages of major compounds compared to the Italian one. In our experiment, *R*. *chalepensis* EO contains higher amounts of 2-nonanone and 2-nonyl acetate (56.73 and 14.16%, respectively) and a lower relative percentage of 2-undecanone (12.74%), which, for the Turkish ones, was 27.9, 10.6, and 43.2%, respectively. Similarly, the EO extracted from Tunisian cultivated *R*. *chalepensis* contained a lower relative percentage of 2-nonanone (20.5%) and a higher percentage of 2-undecanone (39.3%)^36^, compared to the EO from wild plants utilized in this experiment. Such differences in the chemical composition of EOs from the same plant species are expected and probably due to the different geographical area, growth stage and period of harvesting of the plant^37,38^. The evolution of different chemotypes of plant species may represent a form of adaptation to the environment and can be considered as an advantage aimed at avoiding the development of resistance by insects^39^. Dris *et al*.^40^ found that the EO composition of Algerian *L*. *dentata*, that showed a good larvicidal activity against *Culiseta longiareolata* (Macquart) and *Culex pipiens* L. (Diptera Culicidae), was dominated by α-terpinolene, a monoterpene hydrocarbon not detected in the *L*. *dentata* EO of the present study, followed by a much lower relative percentage of camphor. In line with our findings, *A*. *verlotiorum* EO main compound, chrysanthenone, was also reported as a major compound of *Achillea millefolium* L. (Asteraceae). It exhibited significant toxicity rates against *Ae*. *albopictus* and *Cx*. *pipiens*, thus confirming the mosquitoes repellent effect exerted by this compound, even when present in another species^41^.

In the present work, besides the chemical and sensorial characteristics, we described the repellent and toxic activities of *A*. *verlotiorum*, *L*. *dentata* and *R*. *chalepensis* EOs against *Ae*. *albopictus*. Results showed that *A*. *verlotiorum* and *R*. *chalepensis* EOs were able to significantly deter *Ae*. *albopictus* oviposition in open field with a reduction of the oviposition of 61.78 and 81.61%, respectively. In line with our results, other botanical compounds have been recognized as oviposition deterrents against mosquito species. Benelli *et al*.^42^ found that neem extracts were able to deter *Ae*. *albopictus* oviposition up to 88.59% over control. Similarly, *Artemisia annua* L. (Asteraceae) crude extract at 200 ppm exerted oviposition repellence rates against *Ae*. *aegypti*, *Anopheles sinensis* Wiedemann and *Culex quinquefasciatus* Say (Diptera Culicidae) of 43.38%, 73.85% and 41.57% respectively^43^, and the ethanolic extract of *Cassia obtusifolia* L. (Leguminosae) leaves showed a strong repellence against the mosquito *Anopheles stephensi* Liston (Diptera Culicidae) at the dose of 100 mg L^−1^ achieving 75.5% of effective oviposition reduction^44^.

Beside the oviposition deterrence, *A*. *verlotiorum*, *L*. *dentata*, and *R*. *chalepensis* EOs showed larvicidal activity against *Ae*. *albopictus*, with *R*. *chalepensis* that was significantly more toxic than the other two EOs. The toxicity of *R*. *chalepensis* EO tested in this work was, however, lower than the one of the EO extracted from Tunisian cultivated *R*. *chalepensis* plants (LC_50_ = 33.18 μL L^−1^)^36^ but, overall, in the range of the LC_50_ values (LC_50_ range = 11–194 μL L^−1^) reported for 22 plant species EOs against *Ae*. *albopictus* larvae in previous works (reviewed by Pavela^45^).

The dose-response repellence bioassays showed that all the EOs tested had a relevant activity against *Ae*. *albopictus* females. Among the three EOs tested, *R*. *chalepensis* EO was the most effective one, resulting about two times more effective than *L*. *dentata* EO and four time more effective than *A*. *verlotiorum* EO (see Table 4). In general, our results are in line with the repellence against *Ae*. *albopictus* reported for other aromatic plants EOs. Conti *et al*.^46,47^, evaluating the EOs of *Hyptis suaveolens* L., *Salvia dorisiana* Standl., *Salvia longifolia* Nutt., and *Salvia sclarea L*. (Lamiaceae), found RD_50_ values ranging between 0.4 and 1.0 nL cm^−2^. Similar efficacy against *Ae*. *albopictus* was also found for *Coriandrum sativum* (Apiaceae) (RD_50_ = 0.2 nL/cm^2^ skin)^48^ and for Tunisian cultivated *R*. *chalepensis* EOs (RD_50_ = 0.2 nL/cm^2^ skin)^36^.

EOs high volatility usually limits the duration of their efficacy^49^. For this reason, beside the effective dose, it is important to determine the EOs protection time in order to assess their possible practical use as mosquito repellents. *A*. *verlotiorum*, *L*. *dentata* and *R*. *chalepensis* EOs, besides low effective doses, also showed a very good persistency of the repellent effect that, in terms of complete protection time and residual effect, was similar or longer than the DEET one. Experiments on the lasting of the EOs repellence against *Ae*. *albopictus* are scarce. However, in line with our results, Conti *et al*.^47^ found that *S*. *dorisiana*, *S*. *longifolia*, and *S*. *sclarea* EOs were able to completely repel *Ae*. *albopictus* for times ranging from 31 and 21 min at 0.04 μL cm^−2^. Similarly, Das *et al*.^50^ showed that *Curcuma longa* L. (Zingiberaceae), *Pogostemon heyneanus* Benth. (Lamiaceae), and *Zanthoxylum limonella* Alston (Rutaceae) EOs were able to significantly repel laboratory reared *Ae*. *albopictus* mosquitoes up to 23 min at the dose of 5% (about 5 times more concentrate than the solutions tested in the present study). Moreover, Nasir *et al*.^51^, testing *Mentha piperita* L. *Ocimum basilicum* L. (Lamiaceae), and *Zingiber officinale* Rosc. (Zingiberaceae) EOs at 10% (about 10 times more concentrate than the solutions tested in the present study), obtained protection times ranging from 34 to 98 min.

It is noteworthy that the *A*. *verlotiorum*, *L*. *dentata* and *R*. *chalepensis* EOs effective repellent dose was not consistent with the lasting of their effect. *A*. *verlotiorum* EO, although the least effective after the skin treatment, resulted the longer-lasting one among the three tested EOs, assuring at 0.04 μL/cm^2^ skin a complete protection of the treated skin 60% longer than that of the synthetic chemical repellent DEET. On the contrary, *R*. *chalepensis* EO, although the most effective, resulted the less persistent one. Low persistence of *R*. *chalepensis* EO was previously observed also by Conti *et al*.^36^. Such differences in the duration of the effect may also be due to the addictive, synergistic or antagonistic interactions of EOs different compounds, with different volatility^52^.

In this study, *A*. *verlotiorum*, *L*. *dentata* and *R*. *chalepensis* EOs, showing anti-mosquito properties similar or higher than DEET, appear as viable alternative to synthetic repellents. Unlike DEET, EOs based products may be well accepted by the public due to the increasing consumer demand for effective and safe, natural products. More significantly, considering the sensory quality parameters of EOs, the possibility of their use can be expanded by incorporating EOs in sprays, creams, lotions and aerosols based anti-mosquito formulations. The good protection time currently observed can be further increased by polymer encapsulation^53^ and by other compound like vanillin^54^ or by the combined action of optimized EOs mixtures, due to the synergistic co-repellent effect of EOs chemical constituents^50^.

In conclusion, aromatic plants EOs are a valid alternative to synthetic repellents and insecticides for personal and environmental protection against mosquitoes. A new “integrated approach”, deriving from the merging of the repellence test, chemical, and sensorial data, can be useful to identify (or to select among those already existing) new and more appropriate EOs able to ensure the consumers’ compliance and an optimal protection level against mosquitoes.

## Methods

### Plant material, essential oil extraction, and chemical analyses

*A*. *verlotiorum* plants were collected along the river Arno in the Pisa area (Tuscany, Italy), *R*. *chalepensis* in the Northern Apennines (Monte Pisano) (Tuscany, Italy) and *L*. *dentata* in Wadi Thee Ghazal, near Taif (Makkah Province), Saudi Arabia. Plant species were selected among those with floral smell essential oils, the most suitable, in our opinion, to be proposed for the formulation of topical products. In addition, the selected plants are available in many countries with remarkable abundance so their future use in dermatological products seems feasible. Plants were collected at the flowering stage in May 2016, air-dried in the shadow at room temperature, ground and then subjected to hydro-distillation for 2 h in a Clevenger-type apparatus. EOs were collected over water, dried over anhydrous sodium sulphate and stored at 5 °C until analysis. The gas chromatography (GC) analyses were performed as described by Bedini *et al*.^55^. The identification of the constituents was based on the comparison of the retention time with those of authentic samples, comparing their linear indices relative to a series of *n*-hydrocarbons and on computer matching against commercial library of mass spectra^56^ as well as a homemade library of mass spectra built up from pure substances and components of known oils and mass spectra literature data.

### Essential oils smell characterization

The smell profiles of the essential oils were evaluated by a trained panel composed by 13 assessors (“expert panel” of the Department of Agriculture, Food and Environment (DAFE) of University of Pisa). All assessors had previous experience in sensory descriptive analysis^57,58^. The assessors were provided with a specifically developed sensorial sheet consisting of a non-structured, parametric, descriptive scoring chart. The panellists described the main odours characterizing each sample on the basis of defined descriptors (see Tab. 7) ranked on a scale of 0–10 in terms of “Smell intensity”, “Smell persistency”, and “Overall pleasantness” and “Repulsion” as hedonic parameter^59^. Assessors were also asked to provide indications about the possible use (Environmental or Body care) of the EOs. The blind smell test was performed in the morning, in a well-ventilated quiet room and in a relaxed atmosphere. Each panellist was provided with a 2 × 2 cm filter paper soaked with 50 µL of an unknown EO. To avoid cross contamination, the three samples were assessed separately in the same morning (15 minutes waiting between the assessing).

### Essential oils oviposition deterrence

The oviposition deterrence of the EOs was evaluated following the method described by Benelli *et al*.^41^. The experiments were carried out outdoor in an experimental field (about 3,000 m^2^) in Pisa (Italy) from June to September 2017. The EOs were tested at the concentration of 200 ppm in 0.1% Tween 80 tap water solution. For each treatment, four ovitraps filled with 500 mL of EOs solution were paired with four control pots containing Tween 80 0.1% solution only and each pair was separated by 5 m. A Masonite strip (200 × 25 mm) was placed in each pot. The Masonite strips were removed daily for fourteen days and *Ae*. *albopictus* eggs were counted under a stereo microscope. The oviposition activity was expressed as oviposition activity index (OAI): OAI = (NT − NC)/(NT + NC), where NT is the total number of eggs in the test solution and NC is the total number of eggs in the control solution. Positive values (OAI > + 0.3) indicate that the test solutions were attractive. Conversely, negative values (OAI < − 0.3) indicate that the test solutions were deterrent^60^.

### *Ae. albopictus* rearing

Adults of *Ae*. *albopictus* originated from field-collected eggs. For eggs hatching, the Masonite strips were collected daily and placed in 250 mL beakers submerged in tap water under room conditions (26 ± 2 °C, 60% rh, photoperiod of 14:10 h (L:D). For larvicidal treatments, newly emerged larvae were fed with a small amount of cat food until fourth instar. For repellence tests, pupae were then placed in Plexiglas cylindrical cages (diameter 35 cm, length 60 cm) with a cotton stockinet access sleeve on the front. Emerged adults, were maintained (about 300 individuals per cage, sex ratio 1:1) under laboratory conditions, and supplied with 10% sucrose solution.

### Essential oils repellent activity

The repellent effective dose (ED_50_) of the EOs was evaluated using the human-bait technique^61^ with some modifications. Experiments were conducted during the summer of 2017 in the above descripted cages, containing about of 150 field-derived, 8–12 days old females starved for 12 h that had not yet been either blood fed or exposed to any form of repellent. The study was approved by the ethical committee of the University of Pisa (Comitato Bioetico dell’Università di Pisa) and all experiments were performed in accordance with relevant guidelines and regulations. All volunteers provided written informed consent. Ten volunteers were chosen among subjects not allergic to mosquito bites. The volunteers had no contact with perfumed products on the day of the bioassay. After cleaning their hands in distilled water, their forearms were protected with a thick fabric sleeve and the hands with latex gloves, in which a dorsal square area 5 × 5 cm was cut open. The mosquito-exposed skin of one hand was treated with 100 μL of ethanol as a negative control. The other hand was treated with 100 μL of EO ethanolic solution at concentrations varying from 0.00001 to 0.8 µL cm^−2^. After about 5 min, the control hand was inserted inside the cage and exposed to mosquitoes for 3 min. Immediately after, the other hand was treated with the EO solution and, after about 5 min, exposed to mosquitoes in the same cage. The number of probing mosquitoes was recorded by two observers. On rare occasions, when no mosquito attempted to bite the untreated hand, the test was repeated with a new mosquito’s cage. The testing time was between 8:00 and 10:00 a.m. in order to perform the test during the peak of the biting activity of the species

For the evaluation of the protection time, the tests were performed according to Nguyen *et al*.^62^ procedure, with few modifications. Four cages, as above descripted, were assigned to each participant, chosen as above descripted, and randomly used for the experiment. The randomized use of the cages was meant to reduce any bias associated with using only one cage and to prevent accumulation of repellent on or around the single cage, which may bias the evaluation of the effectiveness of the repellents. The experiment was performed as above descripted at the doses of 0.01, 0.02, 0.04, and 0.08 μL cm^−2^ skin. The procedure was repeated at an interval of 15 min. The lasting of the protection from mosquito bites (complete protection time, CPT), was calculated as the time between the application of the repellent and the first of two consecutive mosquito probing attempts. The lasting of the residual protection effect (residual protection time, RPT) was calculated as the time until no more differences in the number of probing were observed between the treated and the control hand. Data used to calculate the protection time were recorded by two observers. 100 μL of 1% *N*,*N*-Diethyl-3-methylbenzamide (DEET) (Sigma-Aldrich, Milan, Italy) ethanolic solution (0.04 μL cm^−2^ skin) was used as positive control, whereas 100 μL of ethanol was used as negative control.

### Essential oils larvicidal activity

Groups of 20 fourth-instar larvae were placed in 250 mL beakers and exposed to 50, 100, 150, 200, 300, 400 and 600 μL L^−1^ of the EOs in 0.1% Tween 80 tap water solutions. As control, 20 larvae were exposed to the 0.1% Tween 80 tap water solution only. Mortality was recorded after 24 h. No food was given to the larvae during the tests^14^. Three replicates for each treatment were performed. Mortality percentage rates were corrected using Abbott’s formula^63^.

### Statistical analysis

Data of the assessments of the smell profiles of EOs and oviposition were processed by one-way ANOVA with the EO treatment as factor. Equality of variances was checked before the analyses by Levene’s test. Means were separated by Tukey’s b post-hoc test. *P* < 0.05 was used as significance level of differences between means. OAI values were evaluated by Kruskal-Wallis test and means separated by pairwise comparison. The distribution of the time to mosquito-landing among EOs treatments and controls was estimated using the Kaplan-Meier method by repeated-measures modelling, with time as a categorical variable. Significant differences between MCPT and MRPT values were determined by Breslow (Generalized Wilcoxon) test^64^. EOs larvicidal activity was evaluated by Log-probit regression. Since no mortality was registered in the control treatment, the larval mortality percentage rates were not corrected. Analysis were performed by the SPSS 22.0 software (IBM SPSS Statistics, Armonk, North Castle, New York, USA).

## Electronic supplementary material

Supplementary information
