# Author Correction: Essential oils sensory quality and their bioactivity against the mosquito *Aedes albopictus*

**DOI:** 10.1038/s41598-019-41665-5

**Published:** 2019-04-11

**Authors:** S. Bedini, G. Flamini, R. Ascrizzi, F. Venturi, G. Ferroni, A. Bader, J. Girardi, B. Conti

**Affiliations:** 1 Department of Agriculture, Food and Environment – University of Pisa, Pisa, Italy; 2 Department of Pharmacy, University of Pisa, Pisa, Italy; 3 Faculty of Pharmacy, Umm Al-Qura University, Makkah, Saudi Arabia

Correction to: *Scientific Reports* 10.1038/s41598-018-36158-w, published online 14 December 2018

The Acknowledgements section in this Article is incomplete.

“The authors wish to thank Mr. Paolo Giannotti for the technical assistance, the panelists and the volunteers who participated to the repellence tests.”

should read:

“The authors wish to thank Mr. Paolo Giannotti for the technical assistance, the panelists and the volunteers who participated to the repellence tests. This research was supported by Italian Ministry of Education, University and Research (MIUR) (PRIN Project 2015 “BIOPIC”, 2015 BABFCF).”

