# Supplementary material for: Essential oils sensory quality and their bioactivity against the mosquito Aedes albopictus
Source: Sci Rep. 2018 Dec 14;8:17857. doi: 10.1038/s41598-018-36158-w (PMC6294827; doi:10.1038/s41598-018-36158-w)
Supplement: Supplementary file 1 — Supplementary information [file 41598_2018_36158_MOESM1_ESM.pdf]

Essential oils sensory quality and their bioactivity against the mosquito *Aedes albopictus*

S. Bedini[1], G. Flamini[2], R. Ascrizzi[2], F. Venturi[1], G. Ferroni[1], A. Bader[3], J. Girardi[1] & B. Conti[1*]

Supplementary information

Table 1S. Chemical composition (%) of *Artemisia verlotiorum, Lavandula dentata* and *Ruta chalepensis* essential oils.

Constituents[a]	*LRI*	*A. verlotiorum*	*L. dentata*	*R. chalepensis*
(E)-2-hexenal	856	0.15	-	-
α-pinene	941	1.19	1.19	0.11
camphene	955	-	1.26	-
thuja-2,4(10)-diene	959	-	0.19	-
sabinene	976	0.27	-	0.32
1-octen-3-ol	980	0.84	-	-
β-pinene	981	-	2.69	0.16
2-octanone	992	-	-	0.38
myrcene	993	1.89	-	-
octanal	1001	0.11	-	-
p-cymene	1027	-	-	0.2
limonene	1032	0.43	0.41	-
1,8-cineole	1035	-	1.93	2.23
(*Z*)-β-ocimene	1042	7.1	-	-
2-heptyl acetate	1045	0.18	-	-
γ-terpinene	1062	-	-	0.26
cis-sabinene hydrate	1070	0.21	-	-
cis-linalool oxide (furanoid)	1076	1.04	-	-
fenchone	1089	-	1.77	-
2-nonanone	1093	-	26.19	-
6,7-epoxymyrcene	1094	0.28	-	-
trans-sabinene hydrate	1095	-	-	56.73
linalool	1101	0.68	-	-
2-nonanol	1101	-	-	3.54
2,6-dimethyl phenol	1108	-	2.2	-
endo-fenchol	1117	0.46	-	-
exo-fenchol	1118	5.19	-	-
chrysanthenone	1127	-	0.35	-
trans-pinocarveol	1141	-	4.19	-
geijerene	1143	34.27	-	-
camphor	1145	-	52.05	-
β-pinene oxide	1158	-	-	1.67
pinocarvone	1163	2.16	-	-
borneol	1167	-	0.36	-

δ-terpineol	1170	-	1.44	-
4-terpineol	1178	0.17	-	-
p-cymen-8-ol	1184	0.71	0.18	0.24
α-terpineol	1191	-	0.36	-
2-decanone	1193	0.4	-	-
myrtenol	1194	-	0.34	-
myrtenal	1195	-	-	1.69
verbenone	1205	-	0.61	-
trans-carveol	1218	-	0.24	-
trans-chrysanthenyl acetate	1235	0.41	-	-
2-nonyl acetate	1237	0.2	-	-
carvone	1244	-	-	14.61
perilla aldehyde	1273	-	0.26	-
*iso*piperitenone	1277	1.01	-	-
pregeijerene	1288	0.17	-	-
2-undecanone	1294	-	-	0.71
2-undecanol	1308	-	-	12.74
eugenol	1358	-	-	1.29
α-copaene	1376	-	-	0.19
β-bourbonene	1384	0.45	-	-
2-dodecanone	1394	0.27	-	-
β-caryophyllene	1420	0.25	-	-
α-humulene	1456	-	-	0.2
(*E*)-β-farnesene	1460	12.62	-	-
2-undecyl acetate	1475	1.31	-	-
γ-muurolene	1477	0.43	-	-
β-selinene	1485	-	-	1.42
bicyclogermacrene	1495	9.9	-	-
2-tridecanone	1496	-	0.36	-
α-alaskene	1512	1.16	-	-
δ-cadinene	1524	-	-	0.15
germacrene D-4-ol	1575	-	0.41	-
spathulenol	1576	0.24	-	-
caryophyllene oxide	1581	0.46	-	-
humulene epoxide II	1606	0.84	-	-
caryophylla-4(14),8(15)-dien-5-ol	1636	3.97	-	-
epi-α-cadinol	1640	0.27	-	-
β-eudesmol	1650	0.61	-	-
selin-11-en-4-α-ol	1653	0.25	-	-
(*Z*)-α-santalol	1681	-	0.22	-
cis-14-nor-muurol-5-en-4-one	1682	2.96	-	-
(Z)-lanceol	1765	0.87	-	-
Total identified		96.38	99.60	98.84

[a], Chemical constituents ≥ 1%; LRI, linear retention index on DB-5 column

Tab. 2S. Protection activity of *Lavandula dentata* EO against *Aedes albopictus* females at different time from the application as skin repellent

Dose[a]	Time[b]								
	**0**	**10**	**20**	**30**	**40**	**50**	**60**	**80**	**90**
**0.01**	88.4±5.9	62.7±19.6	9.9±13.8	0.0±0.0	0.0±0.0	0.0±0.0	0.0±0.0	0.0±0.0	0.0±0.0
**0.02**	97.7±5.1	81.0±10.9	52.5±21.7	35.7±22.3	12.9±13.7	0.0±0.0	0.0±0.0	0.0±0.0	0.0±0.0
**0.04**	100.0±0.0	92.6±9.5	70.8±20.9	42.4±13.9	15.6±14.9	0.0±0.0	0.0±0.0	0.0±0.0	0.0±0.0
**0.08**	100.0±0.0	88.6±2.0	74.9±19.3	55.6±12.6	38.5±25.8	3.2±3.8	0.0±0.0	0.0±0.0	0.0±0.0

[a], (µL/cm^2 of skin); [b], time from the application of the repellent (min). Data are reported as percentage of repellency (number probing untreated hand − number probing treated hand)/number probing untreated hand × 100) ± standard deviation.

Tab. 3S. Protection activity of *Artemisia verlotiorum* EO against *Aedes albopictus* females at different time from the application as skin repellent

Dose[a]	Time[b]								
	**0**	**10**	**20**	**30**	**40**	**50**	**60**	**80**	**90**
**0.01**	75.1±24.5	57.8±26.6	45.7±34.3	22.7±26.5	5.6±7.4	0.0±0.0	0.0±0.0	0.0±0.0	0.0±0.0
**0.02**	94.9±6.0	59.3±23.5	37.3±29.8	25.6±21.1	4.0±6.8	0.0±0.0	0.0±0.0	0.0±0.0	0.0±0.0
**0.04**	99.3±1.7	96.5±7.2	82.4±15.6	69.2±9.5	41.0±15.1	8.0±9.8	0.0±0.0	0.0±0.0	0.0±0.0
**0.08**	100.0±0.0	92.3±5.8	76.7±6.6	71.7±12.0	69.1±4.3	52.3±28.4	31.2±22.4	4.0±9.0	0.0±0.0

[a], (µL/cm^2 of skin); [b], time from the application of the repellent (min). Data are reported as percentage of repellency (number probing untreated hand − number probing treated hand)/number probing untreated hand × 100) ± standard deviation.

Tab. 4S. Protection activity of *Ruta chalepensis* EO against *Aedes albopictus* females at different time from the application as skin repellent

Dose[a]	Time[b]								
	**0**	**10**	**20**	**30**	**40**	**50**	**60**	**80**	**90**
**0.01**	72.8±7.2	62.0±15.0	46.8±13.3	35.9±17.2	7.4±7.4	0.0±0.0	0.0±0.0	0.0±0.0	0.0±0.0
**0.02**	95.3±3.5	75.3±12.5	62.3±12.9	39.0±1.8	12.7± 10.4	0.0±0.0	0.0±0.0	0.0±0.0	0.0±0.0
**0.04**	94.2±2.0	83.7±8.3	68.7±3.5	43.3±21.9	31.95±17.5	12.5±11.1	0.0±0.0	0.0±0.0	0.0±0.0
**0.08**	98.0±1.5	86.1±8.8	78.3±9.3	60.9±8.7	49.8±22.2	30.5±15.5	19.8±14.0	0.0±0.0	0.0±0.0

[a], (µL/cm^2 of skin); [b], Time from the application of the repellent (min). Data are reported as percentage of repellency (number probing untreated hand − number probing treated hand)/number probing untreated hand × 100) ± standard deviation.
